# Folded or Not? Tracking Bet v 1 Conformation in Recombinant Allergen Preparations

**DOI:** 10.1371/journal.pone.0132956

**Published:** 2015-07-17

**Authors:** Felix Husslik, Kay-Martin Hanschmann, Ariane Krämer, Christian Seutter von Loetzen, Kristian Schweimer, Iris Bellinghausen, Regina Treudler, Jan C. Simon, Lothar Vogel, Elke Völker, Stefanie Randow, Andreas Reuter, Paul Rösch, Stefan Vieths, Thomas Holzhauser, Dirk Schiller

**Affiliations:** 1 Division of Allergology, Paul-Ehrlich-Institut, Langen, Hesse, Germany; 2 Division of EU Cooperation/Microbiology, Paul-Ehrlich-Institut, Section Biostatistics, Langen, Hesse, Germany; 3 Department of Biopolymers, University of Bayreuth, Bayreuth, Bavaria, Germany; 4 Department of Dermatology, University Medical Center, Mainz, Rhineland-Palatinate, Germany; 5 Department of Dermatology, Venerology and Allergology, University of Leipzig, Leipzig, Saxony, Germany; Aligarh Muslim University, INDIA

## Abstract

**Background:**

Recombinant Bet v 1a (rBet v 1a) has been used in allergy research for more than three decades, including clinical application of so-called hypoallergens. Quantitative IgE binding to rBet v 1a depends on its native protein conformation, which might be compromised upon heterologous expression, purification, or mutational engineering of rBet v 1a.

**Objective:**

To correlate experimental/theoretical comparisons of IgE binding of defined molar ratios of folded/misfolded recombinant Bet v 1a variants and to determine accuracy and precision of immuno- and physicochemical assays routinely used to assess the quality of recombinant allergen preparations.

**Methods:**

rBet v 1a and its misfolded variant rBet v 1a_S112P/R145P_ were heterologously expressed and purified from *Escherichia coli*. Structural integrities and oligomerisation of the recombinant allergens were evaluated by ^1^H-nuclear magnetic resonance (^1^H-NMR), circular dichroism (CD) spectroscopy, and dynamic light scattering (DLS). IgE binding of defined combinations of rBet v 1a and rBet v 1a_S112P/R145P_ was assessed using immunoblotting (IB), enzyme-linked immunosorbent assay (ELISA) and mediator release (MR) of humanized rat basophilic leukemia cells sensitized with serum IgE of subjects allergic to birch pollen. Experimental and theoretically expected results of the analyses were compared.

**Results:**

^1^H-NMR spectra of rBet v 1a and rBet v 1a_S112P/R145P_ demonstrate a native and highly disordered protein conformations, respectively. The CD spectra suggested typical alpha-helical and beta-sheet secondary structure content of rBet v 1a and random coil for rBet v 1a_S112P/R145P_. The hydrodynamic radii (R_H_) of 2.49 ± 0.39 nm (rBet v 1a) and 3.1 ± 0.56 nm (rBet v 1a_S112P/R145P_) showed monomeric dispersion of both allergens in solution. Serum IgE of birch pollen allergic subjects bound to 0.1% rBet v 1a in the presence of 99.9% of non-IgE binding rBet v 1a_S112P/R145P_. Immunoblot analysis overestimated, whereas ELISA and mediator release assay underestimated the actual quantity of IgE-reactive rBet v 1a in mixtures of rBet v 1a/rBet v 1a_S112P/R145P_ with a molar ratio of rBet v 1a ≤ 10%.

**Conclusion:**

Valid conclusions on quantitative IgE binding of recombinant Bet v 1a preparations depend on the accuracy and precision of physico- and immunochemical assays with which natively folded allergen is detected.

## Introduction

The major allergen Bet v 1 is the main cause of birch pollen allergy affecting millions of patients in Central and Northern Europe [1]. The molecular structures of several recombinant Bet v 1 (rBet v 1) isoforms and variants have been resolved via nuclear magnetic resonance (NMR) spectroscopy and X-ray crystallography [2–6]. Heterologously expressed and purified Bet v 1 has been used in a variety of research applications. It has been tested in animal models to generate optimized allergen immunotherapy (AIT) antigens [7–10]. Moreover, in a number of studies recombinant Bet v 1 variants harboring single or multiple amino acid substitutions were generated to identify clinically relevant B and T cell epitopes and to analyze cross-reactivity of homologous allergens from plant food [4, 11–17]. Furthermore recombinant Bet v 1 variants and fusion proteins are generated and tested as potential hypoallergenic candidates for birch-specific immunotherapy and vaccination [18–25]. Recombinant Bet v 1 is also used to study cellular aspects of Bet v 1-mediated allergies [26–28]. Studies showing comparable allergenic potential of native and recombinant Bet v 1 and addressing the quality of recombinant Bet v 1 preparations were carried out [29–32]. Threshold levels of serum IgE specific for rBet v 1 have been suggested to predict symptoms of birch pollen allergy [33,34]. Furthermore GMP-produced recombinant Bet v 1 preparations including an unfolded variant of Bet v 1 have been used in clinical trials for AIT and several patents on the production of recombinant Bet v 1 for AIT are registered [35]. Thus protocols for heterologous expression of recombinant Bet v 1 in the host *Escherichia coli* are well established and rBet v 1 is also available as allergen reference standard from the European Directorate for the Quality of Medicines and Health Care, EDQM [36].

Physico- and immunochemical integrity and native-like protein conformation are prerequisites to evaluate suitability of rBet v 1 as an allergen biomarker for diagnosis and therapy of birch pollen-related allergy. In general, allergens of the Bet v 1 protein family mostly bind IgE only in their native protein conformation. However, heterologous expression and purification of rBet v 1 might produce a quantitatively unknown fraction of misfolded and thus non-functional (non-IgE binding) rBet v 1. In this regard it has been recently shown that protein conformation of rBet v 1 and thus IgE binding capacity is greatly affected by the conditions used for protein purification [37,38]. Ignorance of these phenomena might lead to false estimates and correlations of the IgE binding potency of rBet v 1 preparations. In case of unfolded hypoallergenic Bet v 1 for AIT it has to be ensured that no folded material is present and that the structural modification is stable over time and IgE reactivity is not re-established. Therefore, attempts have been made to develop immunoassays that distinguish between folded and unfolded variants of Bet v 1 [39].

These observations prompted us to systematically analyze the impact of misfolded rBet v 1 on quantitative IgE binding of rBet v 1 preparations. For this purpose we generated and purified rBet v 1a_S112P/R145P_, a variant harboring two proline residues which impair proper folding into the Bet v 1-type protein conformation. We used compositional mixtures of rBet v 1a, the major allergen isoform of birch pollen, and its derivative rBet v 1a_S112P/R145P_ in defined molar ratios and tested them in immuno- and physicochemical assays routinely used for allergen characterization. Finally we correlated the experimental results with the theoretically expected values for the combinations of the two rBet v 1 proteins in order to i) correlate experimental and theoretically expected data and analytical resolution and to ii) determine the extent of variation depending on biological and technical replicates.

## Material and Methods

### Study design

Three independent preparations of rBet v 1a and rBet v 1a_S112P/R145P_, were made to generate 3 identical compositional mixtures with defined ratios of the two proteins to test both i) the influence of biological replicates and ii) the influence of method variation (technical replicates) on immuno-and physicochemical assays used in allergen characterization. Furthermore we wanted to define the range of quantitative variation within one method to evaluate the correlation of experimental and theoretical data. Finally we compared the resolution of the individual methods to evaluate the performance of the assays with respect to distinguish structural and IgE binding differences in the presence of various ratios of natively folded and unfolded allergen.

### Patients

10 allergic patients suffering from rhinoconjunctivitis or asthma to early flowering tree pollen were included as serum donors. Specific sensitization was documented by positive skin prick test responses and by detection of allergen-specific IgE to either rBet v 1a of 13.7–67.3 kU_A_/L (patients 1–4) or to birch pollen of 83.7->100 kU_A_/L (patients 5–10) as measured by ImmunoCAP^TM^ (Thermo Fisher Scientific, Uppsala, Sweden). Patients were recruited at the University Medical Center Mainz, Department of Dermatology, Mainz, Germany, and at the Klinik für Dermatologie, Venerologie und Allergologie, University of Leipzig, Germany. Serum donations were approved by the local ethics committees of the clinical centers, namely the “Ethik-Kommission an der Medizinischen Fakultät der Universität Leipzig” and the “Ethikkomitee der Universitätsmedizin Mainz”. Study participants provided written informed consent and ethics approval included consent form and consent procedure. Serum from a non-allergic subject was used as negative control for the specific IgE measurements.

### Generation and purification of recombinant Bet v 1a

The open reading frame of Bet v 1a optimized for codon usage of *Escherichia coli* was purchased from Geneart (Life Technologies, Thermo Fisher Scientific, Waltham, MA, U.S.A.) and cloned into bacterial expression vector pET15b (Novagen, Merck, Darmstadt, Germany) using the Clontech InFusion Kit (Mountain View, CA, U.S.A.). Bet v 1a variant rBet v 1a_S112P/R145P_ was generated with the QuickChange Multi Lighting mutagenesis kit (Agilent Technologies, Inc., Santa Clara, CA, U.S.A.) using the Bet v 1a ORF in pET15b_Bet v 1a as DNA template and two mutagenic primers Bet v 1_S112P_ (acaccggatggtggtcccattctgaaaattagc) and Bet v 1_R145P_ (ggtgaaaccctgctgcctgcagttgaaagctat) according to manufacturer’s instructions. The genes were expressed in *E*. *coli* strain BL21(DE3) cells, growing at 37°C in 1 liter of LB medium, containing 50 μg/ml carbenicillin to an OD_600_ value of 0.7. A final concentration of 1 mM IPTG was added to induce protein expression. The cells were harvested 4 h after induction by centrifugation (10000 g, 15 min, 4°C), and the cell pellets were stored at −20°C. Upon expression rBet v 1a was found in both cytosolic soluble and in insoluble fractions (inclusion bodies) of bacterial lysates whereas rBet v 1a_S112P/R145P_ was exclusively found in inclusion bodies. To ensure comparability of protein preparation rBet v 1a and rBet v 1a_S112P/R145P_ were purified from inclusion bodies. The proteins were regained from protein pellets after cell lysis with 10 mM potassium phosphate (KP_i_), pH 7.4, 500 mM NaCl, 8 M urea and 10 mM imidazole, bound to Ni-NTA Superflow (QIAGEN, Hilden, Germany) and refolded by subsequently lowering the urea concentration in 10 mM KP_i_; pH 7.4, 500 mM NaCl, 10 mM imidazole during liquid chromatography. Proteins were eluted by gradual increase of imidazole to 500 mM. Fractions containing the respective protein were pooled and 6xHis-tag was cleaved off with Thrombin (Sigma Aldrich Chemie GmBH, Steinheim, Germany). Proteins were further purified by size-exclusion chromatography (10 m KP_i_, pH 7.4), concentrated, frozen in liquid nitrogen and stored at -80°C. Protein concentrations were determined with bicinchoninic acid using bovine serum albumin as standard (BCA™ protein assay kit, Thermo Scientific, Rockford, IL, U.S.A.).

Three preparations each of rBet v 1a and rBet v 1a_S112P/R145P_ were generated to yield 3 individual rBet v 1a/rBet v 1a_S112P/R145P_ combinations which were analyzed with 1 (immunoblot, CD), 2 (MR assay), and 3 (ELISA) technical replicates each.

### Circular dichroism spectroscopy

Far UV circular dichroism (CD) spectra of the rBet v 1a variants were acquired at 293 K using a Jasco J-810 spectropolarimeter (Japan Spectroscopic, Gross-Umstadt, Germany) at a band width of 1 nm and a sensitivity of 100 mdeg in a 0.2 cm cell. All proteins were analyzed at a concentration of 10 μM in 10 mM KP_i_, pH 7.4. Each measurement comprised the average of 10 repeated scans between 255 and 185 nm.

The mean estimates of residual ellipticities (ΔΘ_experimental_) of rBet v 1a and rBet v 1a_S112P/R145P_ at wave lengths characteristic for secondary structure (193 and 222 nm for α-helix [40], 195 and 218 nm for β-sheet [41], and about 200 nm for random coil [42] were determined according to the following equation:

$$\Delta \Theta_{\text{experimental}}\;=\;\Theta_{x\%\;Betv1a}-\;\Theta_{100\%\;Betv1a\left(S112P/R145P\right)}$$ (1)

Where Θ_x%Bet v1a_ is the residual mean ellipticity of the *rBet v 1a/rBet v 1a*_*S112P/R145P*_ *mixture with 100*, *80*, *60*, *40*, *20*, *10*, *1*, *0*.*1*, *0*.*01*, *and 0% of rBet v 1a*, *respectively and* Θ_100%Bet v1a(S112P/R145P)_ is the residual mean ellipticity of the non-folded rBet v 1a variant. Theoretical mean estimates of residual ellipticities (ΔΘ_theoretical_) were obtained by multiplication of ΔΘ_experimental_ determined with 100% rBet v 1a with 1.25, 1.67, 2.5, 5, 10, 100, 1000, or 10000 representing 80, 60, 40, 20, 10, 1, 0.1, 0.01, and 0.001% of rBet v 1a in the protein mixtures, respectively. The following formula was used:

$$\Delta \Theta_{\;theoretical}=\;\;\Delta \Theta_{\;experimental\;\left(100\%\;rBetv1a\right)}*\;x$$ (2)

where ΔΘ_experimental (100% rBet v 1a)_ is the experimentally determined mean residual ellipticity of 100% rBet v 1a and x is the respective multiplication factor as described above. The experimental/theoretical comparisons for each rBet v 1a/rBet v 1_S112P/R145P_ mix were calculated by dividing ΔΘ_experimental_ by the corresponding ΔΘ_theoretical_.

### Dynamic light scattering

Dynamic light scattering analysis was used to determine hydrodynamic radii (R_H_) in nm using a Zetasizer Nano ZS (Malvern, Herrenberg, Germany). 10 μM of recombinant Bet v 1a and Bet v 1a_S112P/R145P_ in 10 mM KP_i_, pH 7.4 were analyzed at 25°C. Three individual measurements (3 x 10 runs per measurement) per protein were carried out. Data were analyzed by the proprietary software of the DLS instrument where the hydrodynamic radius (R_H_) is calculated from the diffusion coefficient (D) which is obtained by measurement of fluctuations of intensities of scattered light over time and fitting of the respective correlation curve to an exponential function from which the diffusion coefficient can be calculated. R_H_ is then calculated by the Stokes-Einstein Eq (3):

$$R_{\text{H}}=\;\frac{kT}{3\pi \eta D}$$ (3)

Where R_H_ is the hydrodynamic radius, k is the Boltzmann constant, T is the absolute temperature, η is the solvent viscosity, and D is the diffusion coefficient.

### NMR spectroscopy

NMR samples were prepared in 20 mM sodium phosphate buffer, pH 7.0, 0.04% sodium azide and 10% D_2_O containing 30 μM of rBet v 1a or rBet v 1a_S112P/R145P_. Standard 1D ^1^H spectra with WATERGATE solvent suppression [43] were recorded on a Bruker Avance 700 MHz spectrometer at 298 K. NMR data were processed and visualized with the Bruker spectrometer software TopSpin.

### SDS-PAGE and immunoblot analysis

SDS-PAGE was performed with 15% separating gels and 5% stacking gels using a discontinuous buffer system [44]. For immunoblot analysis, 0.5 μg/cm (total protein) of recombinant Bet v 1a/Bet v 1_S112P/R145P_ was transferred onto 0.2 μm nitrocellulose membranes by semi-dry blotting at 0.8 mA/cm^2^ for 1h [45]. After blocking with Tris-buffered saline (TBS) containing 0.3% Tween 20, blots were incubated overnight at room temperature with 10 μl of human serum pool in 1 ml TBS containing 0.05% Tween 20 and 0.1% BSA. After extensive washing, blotting membranes were incubated for 1h with horseradish peroxidase labelled mouse anti-human IgE antibody (Clone B3102E8, Southern biotech via Biozol, Eching, Germany), diluted to 1:100000. IgE-binding proteins were visualized by chemiluminescence (LumiGLO Reserve diluted 1:3, KPL via Medac, Wedel, Germany). For detection of IgG binding to rBet v 1a variants on nitrocellulose membranes, Bet v 1-specific polyclonal rabbit IgG (ALK, Hørsholm, Denmark) was diluted 1:10000 in TBS containing 0.05% Tween 20 and 0.1% BSA and incubated for 1 h at room temperature. Bound IgG was detected with goat anti-rabbit IgG conjugated with horseradish peroxidase (Southern biotech via Biozol, Eching, Germany) (1:10000 dilution) as described above.

IgE bound to rBet v 1 in Western blot analyses was analyzed by densitometry (Fusion Fx, Vilber Lourmat, Eberhardzell, Germany) and evaluated using ImageJ (Rasband, NIH, Bethesda, MD, U.S.A.) Theoretical mean estimates (me_theoretical_) of IgE signals after 100 ms exposure were calculated by setting the IgE signal of rBet v 1a to 100% and the IgE signals of the rBet v 1a/rBet v 1a_S112P/R145P_ combinations to 80, 60, 40, 20, 10, 1, 0.1, 0.01, 0.001, and 0% according to the respective molar content of rBet v 1a in the protein mixtures. The following equation was used:

$$me_{theoretical}=\;me_{\text{experimental }\left(100\%\;rBet\;v\;1\;a\right)}\;*\;\frac{x}{100}$$ (4)

where me_exp(100% Bet v 1a)_ is the densitometric IgE signal of 100% rBet v 1a, x is mol% (i.e. 80, 60, 40, 20, 10, 1, 0.1, 0.01., 0.001, or 0%) of rBet v 1a in the respective rBet v 1a/rBet v 1a_S112P/R145P_ mixture, and me_theoretical_ is the resulting calculated mean estimate of the IgE signal in dependence of rBet v 1a content. The experimental/theoretical comparisons for each rBet v 1a/rBet v 1a_S112P/R145P_ mix was calculated by dividing the experimentally derived mean estimate (me_experimental_) by the respective me_theoretical_ and listed in S2 Table. Immunoblot data were statistically analyzed assuming a linear quantitative correlation of IgE signal and amount of protein blotted.

### Inhibition IgE ELISA

For IgE-ELISA inhibition experiments, Nunc Maxisorp plates (Fisher Scientific, Schwerte, Germany) were coated overnight at room temperature with 50 ng Bet v 1/100 μl of 10 mM potassium phosphate-buffered saline (PBS). After blocking with PBS containing 2% BSA, plates were incubated with the human serum pool (dilution 1:80) and increasing molar ratios of rBet v 1a/rBet v 1a_S112P/R145P_ for 3 hrs at room temperature in PBS containing 0.05% Tween 20 and 0.1% BSA. Allergen-specific human IgE was detected with a horseradish peroxidase-conjugated mouse anti human IgE antibody (Clone B3102E8, Southern biotech via Biozol, Eching, Germany) diluted 1:1000. As substrate 3,3′,5,5′-tetramethylbenzidine (Roth, Karlsruhe) was used for the horseradish peroxidase and the absorbances at 450 nm and 630 nm were measured after stopping the reaction with 25% H_2_SO_4_. For all data points absorbances at 630 nm were substracted from absorbances at 450 nm. Inhibition of IgE binding was calculated with the following equation:

$$\left(1-\;\frac{abs_{sample}}{abs_{max}}\right)*100\%$$ (5)

Where abs_sample_ is the absorbance of the respective sample and abs_max_ is the absorbance without inhibitor. Experimental half-maximal inhibition (EC_50 experimental_) of IgE binding to rBet v 1a (ELISA) was calculated using a four parameter sigmoid curve fit with the restrictions that slope, minimum and maximum asymptote had to be the same for all curves to ensure parallelism among curves. The following equation was used:

$$F\left(x\right)=\;\frac{a-d}{1+\;\;{\left(\frac{x}{c}\right)}^{b}}+d$$ (6)

where F(x) is the inhibition of IgE binding (%), a is the minimum asymptote, b is the slope, c is the EC_50_, and d is the maximum asymptote. Theoretical half-maximal inhibition (EC_50 theoretical_) was obtained by multiplication of EC_50 experimental_ obtained by 100% rBet v 1a with factors 1.25, 1.67, 2.5, 5, 10, 100, 1000, 10000 representing 80, 60, 40, 20, 10, 1, 0.1, 0.01, and 0.001% of rBet v 1a in the protein mixtures, respectively. The following equation was used:

$$EC_{50\;theoretical}=\;EC_{50\;experimental\;\left(100\%\;rBetv1a\right)}*\;x$$ (7)

where EC_50 experimental (100% rBet v 1a)_ is the experimentally derived half maximal effective concentration with 100% rBet v 1a and x is the respective multiplication factor as described above. The experimental/theoretical comparisons for each rBet v 1a/rBet v 1a_S112P/R145P_ mix was calculated by dividing EC_50 experimental_ by the respective EC_50 theoretical_. For statistical analysis the EC_50_ values with 95% Confidence Intervals were estimated for all curves showing a significant regression and/or having values measured at least from minimum asymptote up to the inflection point of the curve. P values for significant horizontal shifts of the curves, i.e. significant different EC_50_ values were calculated (P values were not adjusted for multiple comparisons due to the exploratory character of the study).

### β-hexosaminidase release from humanized rat basophil leukaemia (RBL) cells

The mediator release assay was performed as described [46]. Briefly, RBL cells expressing the α-chain of the high affinity receptor FcεRI for human IgE were sensitized overnight with a sera pool of birch pollen allergic subjects (diluted 1:40). After washing, cells were stimulated with serial dilutions of rBet v 1a/rBet v 1a_S112P/R145P_ compositional mixtures of defined ratios. Degranulation was quantified by photometric measurement of β-hexosaminidase activity in the culture supernatants. The percentage of β-hexosaminidase activity relative to cells lysed with Triton X-100 (Sigma-Aldrich, Steinheim, Germany) was calculated and corrected for spontaneous release (sensitized cells without allergen). Experimental and theoretical half maximal β-hexosaminidase releases (EC_50 experimental_ and EC_50 theoretical_) were calculated in a similar fashion as described for the ELISA above using the similar formula:

$$F\left(x\right)=\;\frac{a-d}{1+\;\;{\left(\frac{x}{c}\right)}^{b}}+d$$ (8)

Where F(x) is the observed mediator release induced be the respective rBet v 1a/rBet v 1a_S112P/R145P_ mixture. Fit of dose-response curves and statistical analysis was done in a similar fashion as described for ‘Inhibition IgE ELISA’ above.

### Mass spectrometry analysis of recombinant Bet v 1a

We expressed and purified rBet v 1a and rBet v 1a_S112P/R145P_ to homogeneity from *Escherichia coli*. The amino acid sequence of rBet v 1a and rBet v 1a_S112P/R145P_ was confirmed by LC_MS with sequence coverage of 72% and 78% and a score of 11137 and 3957, respectively. The peptides harboring either S112 and R145 or S112P and R145P were individually double checked and detected with a mass error of 1.2 ppm and 2.5 ppm, or 2.4 ppm or 1.9ppm, respectively. The identity of rBet v 1a_S112P/R145P_ was confirmed by liquid chromatography mass spectrometry (LC-MS) after SDS PAGE separation as described elsewhere [47] with slight modifications. Peptides were eluted with 25 mM NH_4_HCO_3_; 10% aceto nitrile (ACN) and the digestion was stopped by adding 5% formic acid. The peptides were analysed by using a nano-ultra performance LC system coupled to a nano-ESI-MS (nano Acquity UPLC nanoESI Synapt-MS, Waters, Milford, US) with a 5 μm symmetry 180 μm x 20 mm c18 pre-column and a 1.7 μm BEH 130 100 μm x 100 mm c18 separation column. A 30 minutes gradient (3% to 40% ACN at 500 nl/minute) after 3 minutes of trapping (99% water at 5 μl/minute) was applied to separate peptides. MS was operated in V mode, acquiring MSE data and applying standard parameters. Data analysis was performed with ProteinLynx Global Server version 2.4 (Waters), searching an in house database consisting of the Uniprot database (as of May 2011, restricted to reviewed entries of eukaryotic organisms) and the amino acid sequences of recombinant Bet v 1a and Bet v 1a_S112P/R145P_. Protein hits were accepted at a false positive rate of less than 4%.

## Results

### Recombinant Bet v 1aS112P/R145P lacks Bet v 1a-type protein conformation

**Fig 1:**
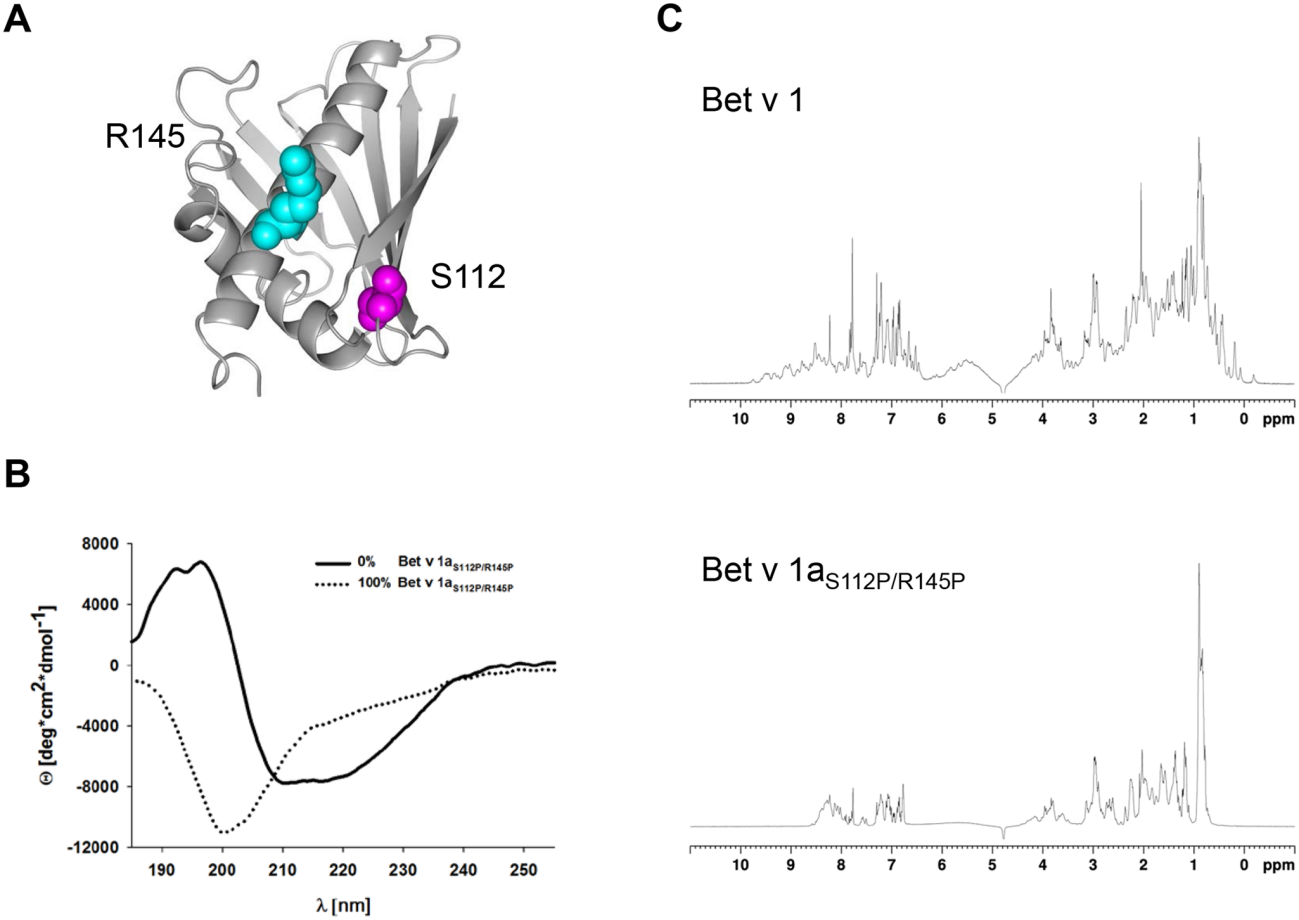
Structural integrity of recombinant Bet v 1a and Bet v 1a_S112P/R145P_. A) Secondary structure topology of Bet v 1a (pdb: 1BV1). The amino acids S112 (purple) of β-strand 7 and R145 (cyan) of α-helix 3 were exchanged for proline. B) Circular dichroism of rBet v 1a and rBet v 1a_S112P/R145P_. C) ^1^H-NMR spectra of 30 μM of rBet v 1a (upper panel), 30 μM rBet v 1a_S112P/R145P_ (middle panel), and the overlay rBet v 1a in red and rBet v 1a_S112P/R145P_ in black (lower panel). 3D-model of Bet v 1a was visualized with PyMOL [54].

To generate a rBet v 1a variant unable to fold into the native Bet v 1-type conformation we substituted prolines for serine 112 and arginine 145, respectively to create rBet v 1a_S112P/R145P_ (Fig 1a). The S112P substitution causes a significant change in the secondary and tertiary structure of Bet v 1a and reduces binding of serum IgE as described previously [48]. Additionally we chose the exchange R145P to prevent a potential formation of the Bet v 1a-typical long extended C-terminal alpha-helix that might contribute to IgE binding of the allergen. When we analyzed the secondary structure of the two proteins by circular dichroism (CD) spectroscopy, we found typical Bet v 1-like spectrum for rBet v 1a, indicating high content of β-pleated sheets and α-helices, whereas rBet v 1a_S112P/R145P_ showed very low ellipticity below 200 nm as characteristic for unstructured protein (Fig 1b). Next we recorded 1D-^1^H-NMR spectra to detect differences in signal dispersion of rBet v 1a versus rBet v 1a_S112P/R145P_ (Fig 1c). rBet v 1a showed its characteristic spectrum with a high degree of resonance dispersion resulting from secondary structural elements like α-helices and β-sheets [49,50]. In contrast, the spectrum of rBet v 1a_S112P/R145P_ exhibited typical random coil shifts close to those found in conformationally disordered peptides [51–53]. We concluded that the amino acid substitutions S112P and R145P abolish the original protein fold of rBet v 1a. To analyze dispersity and potential aggregation of rBet v 1a and rBet v 1a_S112P/R145P_ in solution, we determined the hydrodynamic radii (R_H_) in dynamic light scattering (DLS) and the ^15^N spin relaxation rates of rBet v 1a in NMR. The R_H_ of 2.49 ± 0.39 nm (rBet v 1a) and 3.1 ± 0.56 nm (rBet v 1a_S112P/R145P_) as well as the relaxation rates (S1 File) suggested that both proteins were mono-disperse in solution and did not aggregate.

### Recombinant Bet v 1a_S112P/R145P_ does not bind serum IgE of subjects allergic to birch pollen

**Fig 2:**
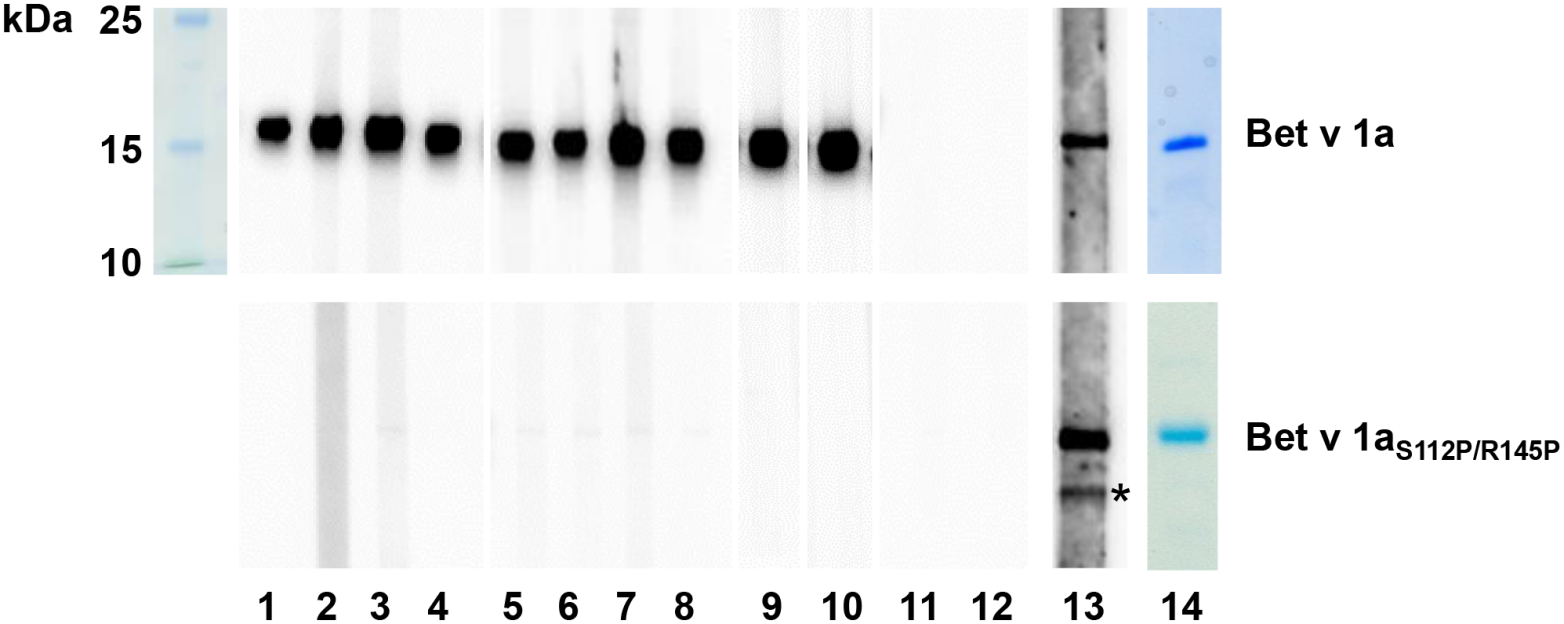
Immunoglobulin binding of rBet v 1a and rBet v 1a_S112P/R145P_. Binding of IgE from sera of subjects allergic to birch pollen bound to purified (lane 14, Coomassie stain) rBet v 1a (upper panel) and rBet v 1a_S112P/R145P_ (lower panel) (lanes 1–10). Binding of rabbit anti-Bet v 1 IgG to the rBet v 1a proteins is shown (lane 13). A minor degradation product of rBet v 1a_S112P/R145P_ detected by IgG is shown (*). As controls, serum of non-allergic subject (lane 11) and buffer control (HRP-conjugated anti human IgE antibody only) (lane 12) were used.

Next we tested antibody binding of the allergen variants in immunoblot analysis (Fig 2). Both proteins bound polyclonal Bet v 1-specific IgG from rabbit serum, showing that antibody binding *per se* is not compromised in rBet v 1a_S112P/R145P_. In contrast no binding of human IgE to rBet v 1a_S112P/R145P_ as opposed to rBet v 1a could be detected, supporting the general view that rBet v 1a binds human IgE only in its native protein conformation. We conclude that lack of IgE binding to rBet v 1a_S112P/R145P_ is due to an irreversible change of the Bet v 1-like protein fold.

### Circular dichroism of rBet v 1a/rBet v 1a_S112P/R145P_ combinations

**Fig 3:**
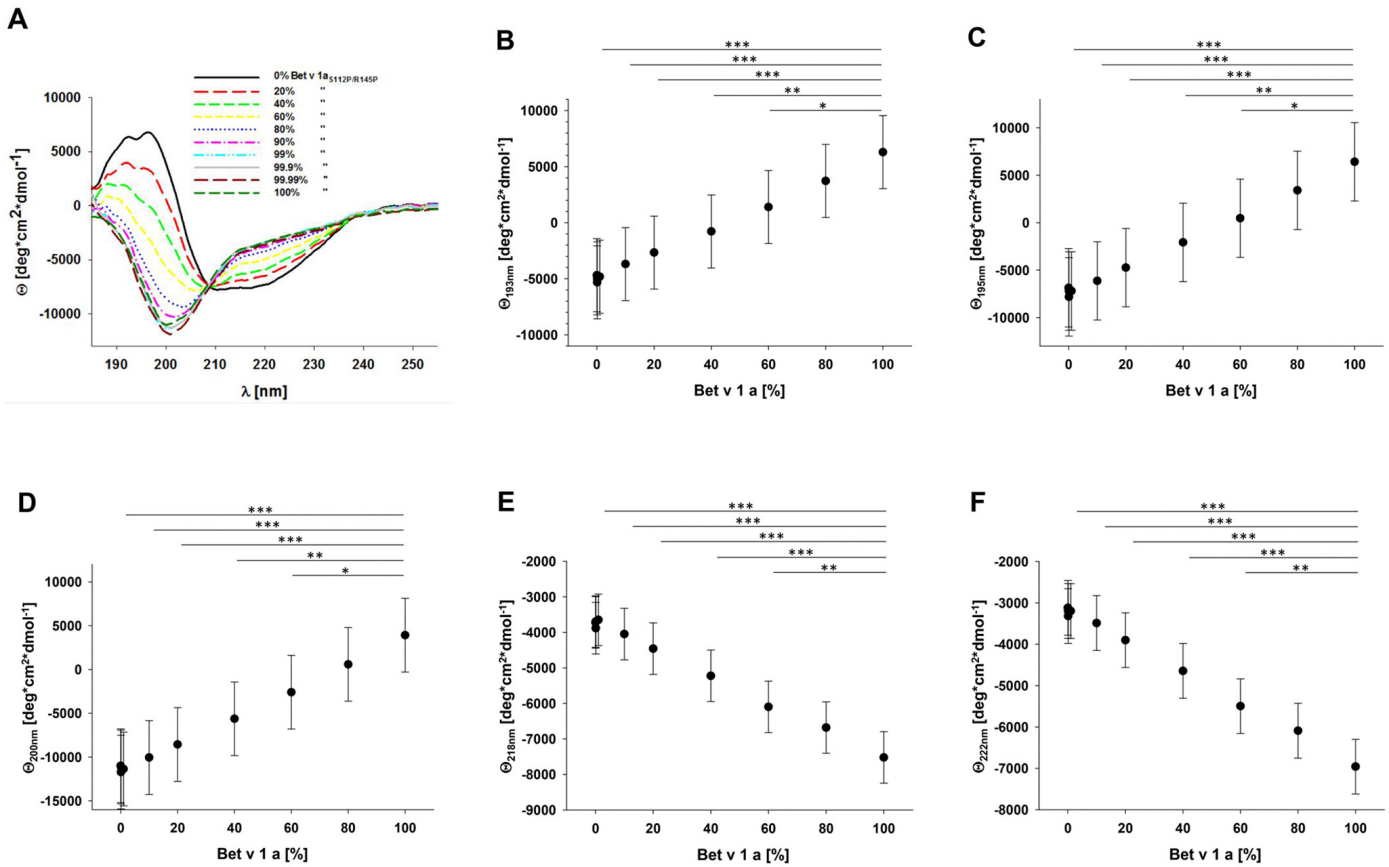
Circular dichroism of rBet v 1a/rBet v 1a_S112P/R145P_ combinations. A) Circular dichroism of defined molar ratios of rBet v 1a/rBet v 1a_S112P/R145P_. 5 μM total rBet v 1a was measured with increasing fractions of rBet v 1a_S112P/R145P_ from 0% to 100%. Mean residual ellipticities with 95% confidence interval and statistical evaluations at wave lengths 193 nm (B), 195 nm (C), 200 nm (D), 218 nm (E), and 222 nm (F) are shown.

Next we analyzed the CD spectra of mixtures of rBet v 1a and rBet v 1a_S112P/R145P_ in defined molar ratios from 100 to 0.01% rBet v 1a to observe the corresponding quantitative changes in ellipticities in particular at 193, 195, 200, 218, and 222 nm, because these wave lengths are most prominent for protein secondary structures (Fig 3). When we compared the experimentally determined mean residual ellipticities with those we theoretically expected, we observed that the experimental-theoretical comparisons correlated well with those calculated for molar combinations from 100% to 10% rBet v 1a (S1 Table). Below 10% of rBet v 1a, however, low linear correlations of the experimental and theoretical ellipticities were observed. According to the 95% confidence interval for each mean residual ellipticity we found that rBet v 1a mixtures containing 60% of rBet v 1a_S112P/R145P_ could be resolved from 0% of the Bet v 1 folding variant at wave lengths 193, 195, 200, and 218 nm, respectively, whereas the ellipticities of the rBet v 1a/rBet v 1a_S112P/R145P_ combinations at 222 nm clearly separated 40% of rBet v 1a_S112P/R145P_ from 0% rBet v 1a_S112P/R145P_ (Fig 3F).

### Immunochemical analyses of rBet v 1a/rBet v 1a_S112P/R145P_ combinations

**Fig 4:**
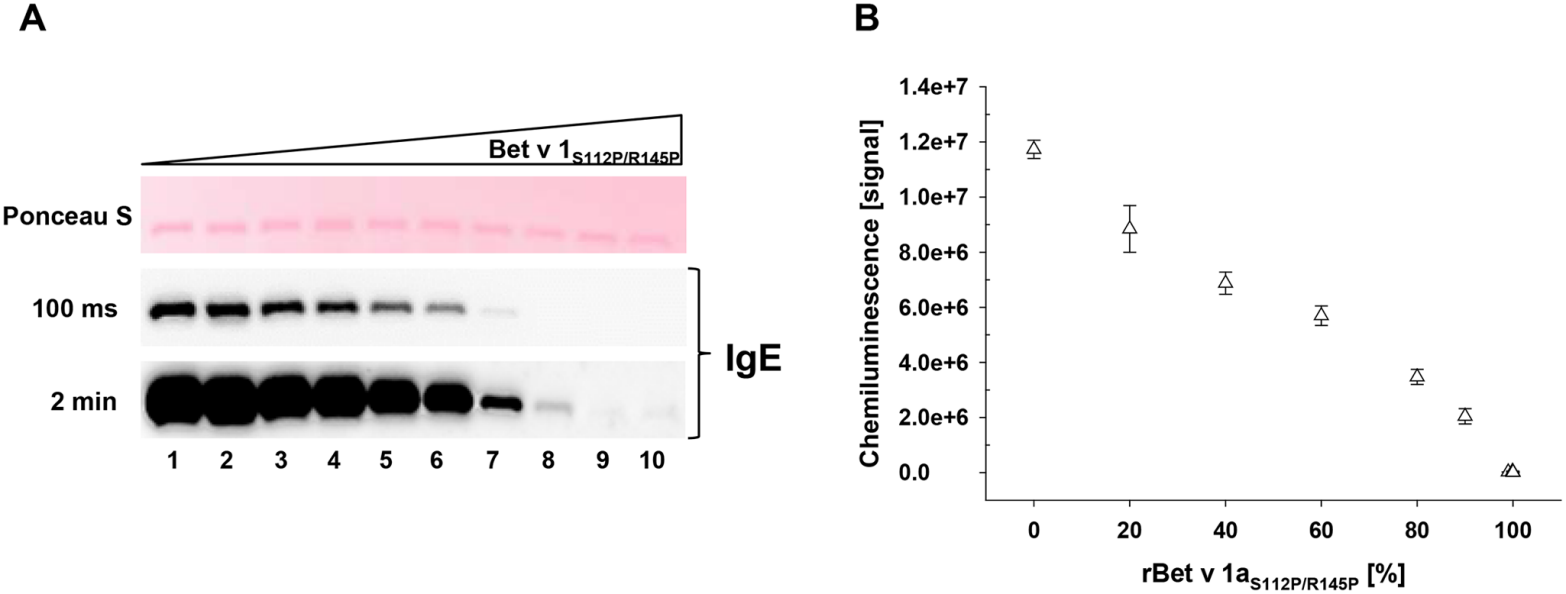
IgE Immunoblot of rBet v 1a/rBet v 1a_S112P/R145P_ mixtures. A) 1 μg of total rBet v 1a with increasing fractions of rBet v 1a_S112P/R145P_ 0% to 100% were transferred onto nitrocellulose and stained with Ponceau S. Binding of sera pool IgE to rBet v 1a combinations was determined by chemiluminescence after 100 milliseconds. To visualize IgE signals with molar ratios of rBet v 1a_S112P/R145P_ from 99% to 100% (lanes 7–10) a 2 min exposure is shown (left). B) Chemiluminescence was quantified densitometrically and plotted against the molar fraction of rBet v 1a_S112P/R145P_ (right).

To correlate the physicochemical data in the previous section with the IgE binding of defined ratios of rBet v 1a and rBet v 1a_S112P/R145P_ we performed a series of immunochemical analyses. First we tested the signal intensity of serum IgE from birch pollen allergic subjects bound to the rBet v 1a protein mixtures in immunoblot (Fig 4). As expected, we observed decreasing IgE signal intensities with increasing molar fraction of unfolded rBet v 1a_S112P/R145P_ in the rBet v 1a/rBet v 1a_S112P/R145P_ combinations. Experimental-theoretical comparison of the IgE signal, however, ranged from 0.94 to 13.16 (80% to 0.01% Bet v 1a), thus over-estimating the quantity of IgE bound to rBet v 1a ≤ 1% in the protein mixtures blotted (S2 Table).

**Fig 5:**
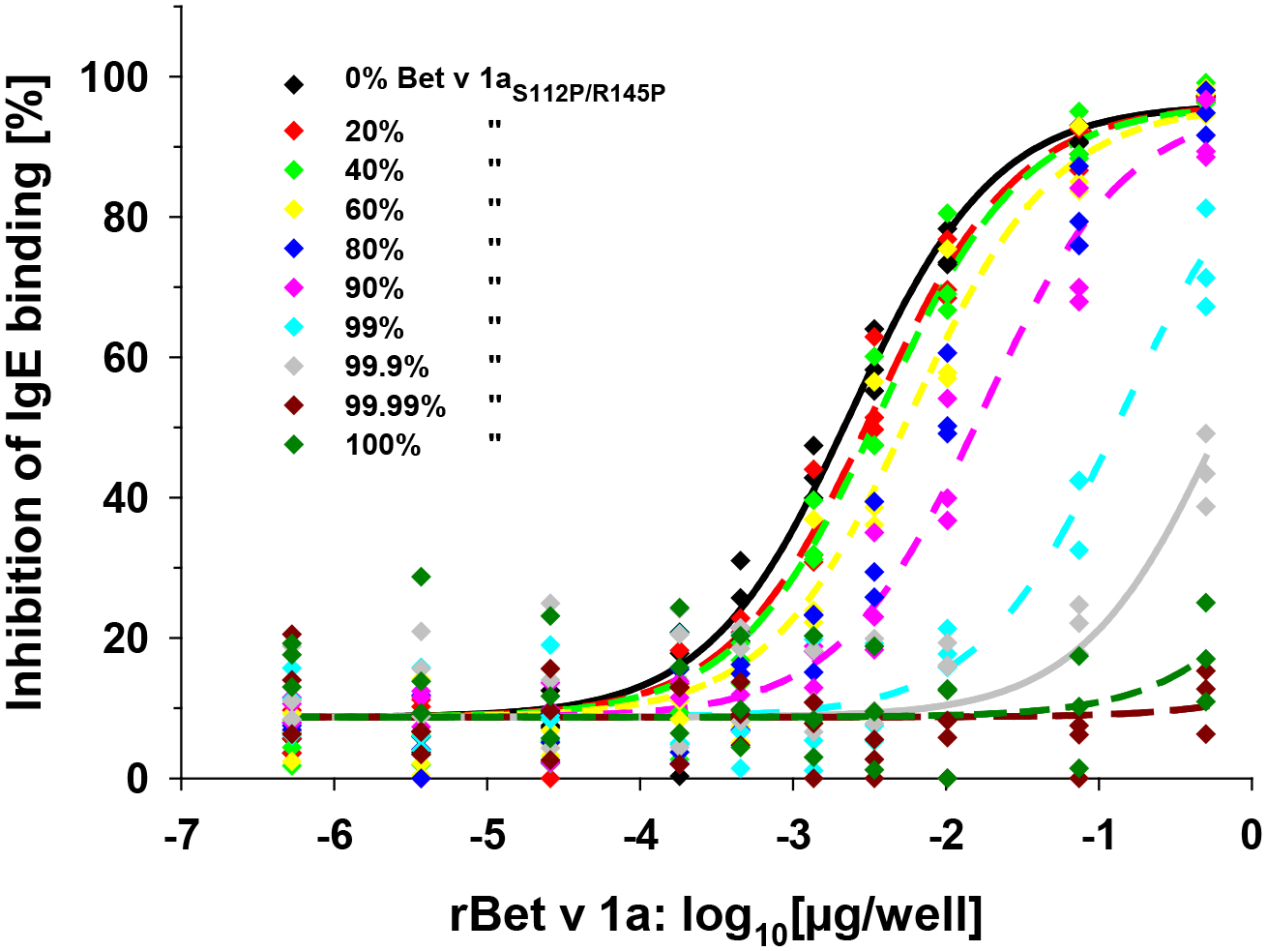
Inhibition of IgE binding to immobilized rBet v 1a. Dose-dependent inhibition of IgE binding to surface-coated rBet v 1a by rBet v 1a/rBet v 1a_S112P/R145P_ mixtures in the presence of increasing molar ratios of rBet v 1a_S112P/R145P_ in ELISA. Curves were fitted in parallel with a 4-parameter, logistic sigmoidal curve fit with same slope, lower and upper asymptote for all curves.

Next we tested inhibition of IgE antibody binding to immobilized rBet v 1a with rBet v 1a/rBet v 1a_S112P/R145P_ protein combinations in ELISA (Fig 5). Since only rBet v 1a is the IgE-reactive component, a rBet v 1a concentration-dependent shift of half-maximal inhibition (EC 50) of IgE binding was expected. We fitted the inhibition curves with a 4-parameter logistic model assuming sigmoidal curve fit with same lower and higher asymptote for all curves. Estimated common slope was 0.91 with a 95% confidence interval of 0.85–0.96. As opposed to rBet v 1a the unfolded rBet v 1a_S112P/R145P_ could not inhibit IgE-Bet v 1a complex formation. The experimental-theoretical values for half-maximal inhibition ranged from 1.09 with 80% of rBet v 1a to 0.29 with 0.1% of rBet v 1a in the protein mix with lower content of IgE-reactive rBet v 1a (<0.01%) yielding non-evaluable inhibition curves (S2 Table). Only half-maximal inhibitions observed with 80% and 99.9% of rBet v 1a_S112P/R145P_ differed statistically significant (p = 0.039 and p = 0.002) from EC_50_ obtained for 100% rBet v 1a.

**Fig 6:**
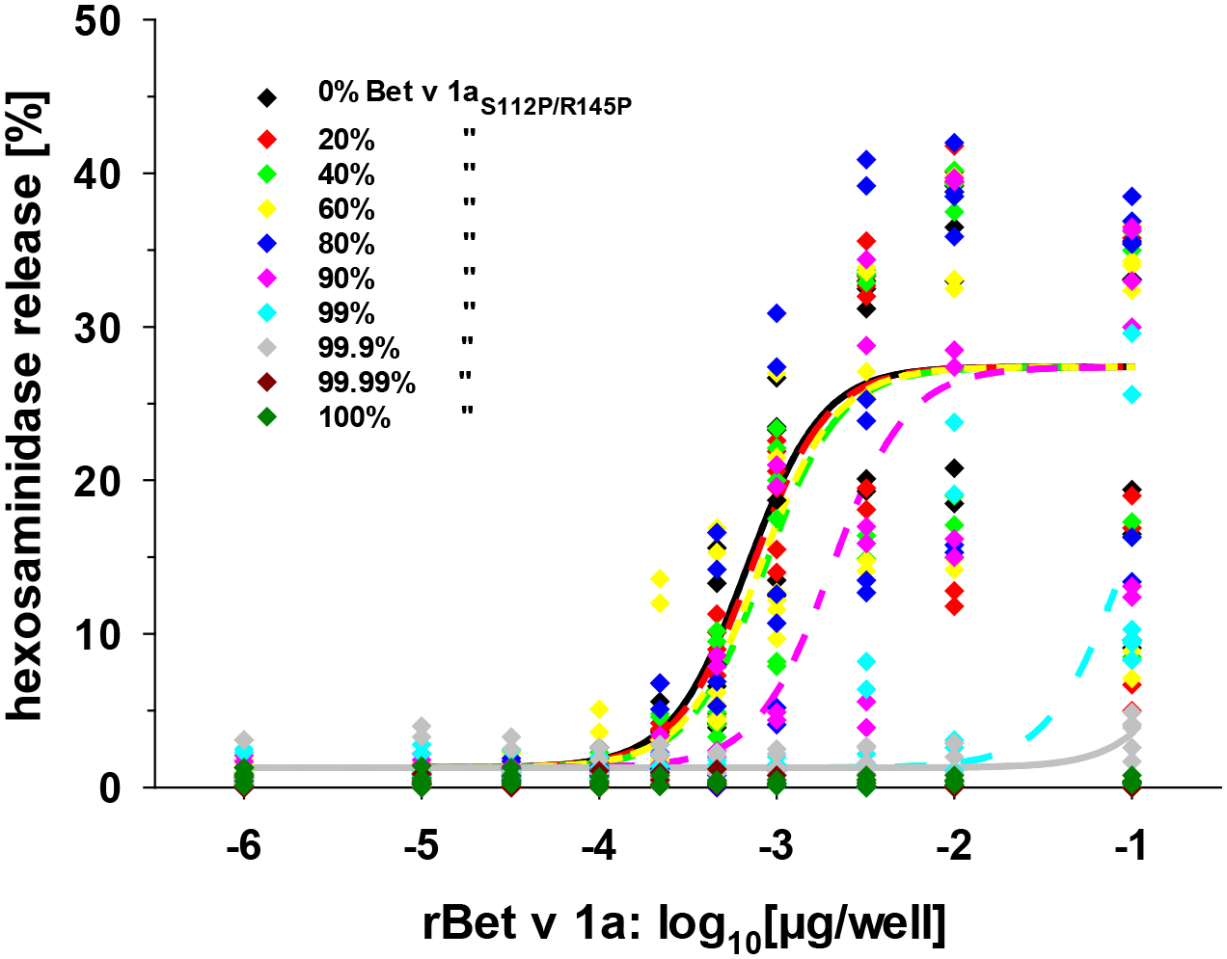
Mediator release of humanized rat basophils. β-hexosaminidase release of humanized rat basophil leukemia cells sensitized with a pool of human sera of donors allergic to birch pollen. Cross-linking of membrane-bound human IgE by IgE-Bet v 1 interaction and subsequent release of β-hexosaminidase was determined in the presence of defined molar ratios of rBet v 1a/rBet v 1a_S112P/R145P_. The legend shows molar ratios (in %) of rBet v 1a_S112P/R145P_ in the rBet v 1a/rBet v 1a_S112P/R145P_ combinations analyzed.

So far the immunoassays above addressed only serological IgE binding to rBet v 1a combinations. To analyze the biological activity of the recombinant allergens in a cellular system we carried out mediator release in humanized rat basophil leukemia cells sensitized with pooled sera IgE (Fig 6). We evaluated the mediator release assays in the same fashion as we did for the ELISA above and found that rBet v 1a/rBet v 1a_S112P/R145P_ protein mixtures induced mediator release up to a rBet v 1a content of 1% with experimental-theoretical values of half maximal mediator releases of 0.86 to 1.32 (20–99% rBet v 1a_S112P/R145P_). However lower contents of IgE binding rBet v 1a (<0.1%) did not allow valid determination of half-maximal mediator release. No significant differences could be observed between the EC_50_ values for 100% rBet v 1a and all rBet v 1a/rBet v 1a_S112P/R145P_ compositional protein preparations. However, according to a confidence interval of 95%, only the rBet v 1a/rBet v 1a_S112P/R145P_ protein combination containing 1% rBet v 1a resolved analytically from the mediator release obtained with 100% rBet v 1a.

**Fig 7:**
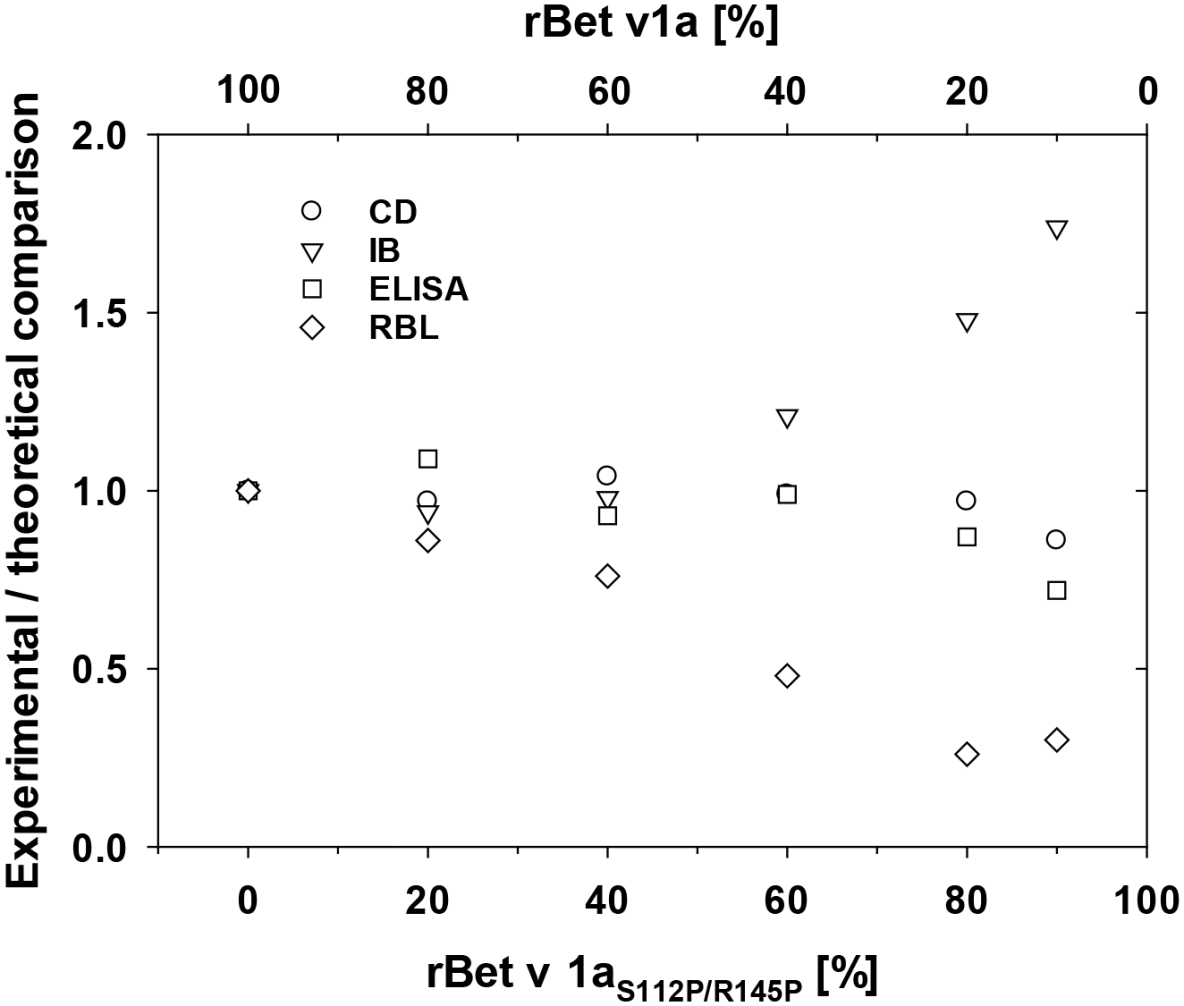
Experimental/theoretical comparison of CD, IB, ELISA and RBL mediator release. The experimental/theoretical comparisons of the individual experiments shown in Figs 3–6 for molar fractions of rBet v 1a_S112P/R145P_ from 0% to 90% in rBet v 1a/rBet v 1a_S112P/R145P_ combinations analyzed are shown.

Finally we compared the experimental/theoretical comparisons of all assays used to analyze rBet v 1a/rBet v 1a_S112P/R145P_ protein combinations with up to 90% of unfolded rBet v 1a_S112P/R145P_ (Fig 7). Whereas the experimental/theoretical values for CD_218nm_ and ELISA were 0.86 and 0.72, respectively, 1.74 and 0.3 were determined for immunoblot and RBL mediator release. The experimental/theoretical comparisons of all assays scattered largely for Bet v 1a/rBet v 1a_S112P/R145P_ protein combinations with molar content of rBet v 1a < 10%.

## Discussion

Recombinant Bet v 1a is used in basic research as well as in in vitro allergy diagnosis and is available as chemical reference standard from the European Directorate for the Quality of Medicines and Health Care (EDQM). In particular for diagnostic purposes, recombinant Bet v 1 needs to bind IgE from sera of subjects allergic to birch pollen in a similar fashion as the native allergen since clinically relevant IgE interaction of Bet v 1 requires a native protein conformation. On the other hand recombinant unfolded Bet v 1 has been used in allergen immunotherapy trials and in this scenario refolding needs to be avoided to ensure the safety of study participants.

The major goal of this study was to correlate changes of secondary/tertiary structure of recombinant variants of Bet v 1a with quantitative IgE binding of protein mixtures comprising defined molar fractions of IgE-reactive natively folded rBet v 1a and non IgE-binding artificially stably unfolded rBet v 1a_S112P/R145P_. We applied physico- and immunochemical assays that are routinely used to judge quality and IgE binding of rBet v 1a preparations according to two quantitative parameters, namely i) the accuracy with which the actual IgE-binding protein moiety is determined and ii) the precision according to the confidence intervals of 95% chosen with which the actual IgE-binding protein moiety is determined resolved quantitatively by the respective analytical method.

All assays showed experimental-theoretical comparisons (0.26–1.74) up to a molar fraction of 10% rBet v 1a in the rBet v 1a/rBet v 1a_S112P/R145P_ protein mixtures. However with molar ratios of rBet v 1a < 10% we found that all assays performed with large deviations from expected values. As opposed to ELISA and cellular mediator release, immunoblot analysis overestimated the actual amount of rBet v 1a-specific bound serum IgE.

Considering the accuracy, precision and resolution according to 95% confidence intervals of the individual assays we found that circular dichroism and ELISA could differentiate a 1.67-fold reduction (i.e. 60% of rBet v 1a in a rBet v 1a/rBet v 1a_S112P/R145P_ mixture) of the total IgE binding content of rBet v 1a (i.e. 100% rBet v 1a) with an accuracy of 1.03 (CD_222nm_) and 0.93 (ELISA), respectively (Table 1). In this regard the immunoblot and cellular mediator release assays performed lower as they could reliably differentiate only a 5-fold (20% mol ratio of rBet v 1a) and 100-fold (1% mol ratio of rBet v 1a) reduction of the IgE binding recombinant allergen, respectively. These findings did not necessarily correlate with the statistical analyses where we found that significant reduction of both IgE binding and native-like Bet v 1a secondary structure in the rBet v 1a/rBet v 1a_S112P/R145P_ mixtures was observed with molar ratios of rBet v 1a of 80% (IB), 20% (ELISA), and 60% (CD_222nm_). No statistical significance was obtained for the cellular in vitro mediator release assay, because of large variation of the results in this biological assay. Our analyses revealed that experimental/theoretical comparisons of physicochemical and immunoassays with physically integer and IgE-reactive rBet v 1a in the range of 100–10% molar content lies within 0.26–1.74, with CD and ELISA correlating best with the theoretical values.

**Table 1:** Accuracy, precision and resolution of methods to distinguish rBet v 1a conformation-dependent IgE binding and secondary structure of rBet v 1a/rBet v 1a_S112P/R145P_ mixtures.

Method	experimental/theoretical comparison	95% CI	p-value	resolution^1^: %	resolution^1^: factor
**CD_222nm_**	1.03	-6157 to -4835 [Θ]^2^	0.0039	60	1.67
**IB**	1.48	1.94*10^6^−6.21*10^6^ [au]^3^	<0.0001	20	5
**ELISA**	0.93	3.05–4.62 [EC_50_]^4^	0.412	60	1.67
**RBL**	1.32	44.4–174.8 [EC_50_]^5^	0.682	1	100

^1^analytical resolution between 100% and x% Bet v 1a in Bet v 1a/Bet v 1a_S112P/R145P_ mixtures

^2^in deg*cm^2^*dmol^-1^

^3^arbitrary units

^4^inhibitor concentration (ng/well) for half-maximum inhibition of IgE binding

^5^protein concentration (ng/well) for half-maximum mediator release

CD_222nm_: mean residual ellipticiy at 222 nm; IB: immunoblot; ELISA: Inhibition-Enzyme-linked immunosorbent assay; RBL: cellular mediator release with humanized rat basophilic leukemia cells

In the European Pharmacopeia Monograph on Allergen Products (01/2010:1063) the total allergenic activity of an allergen product as assayed by inhibition of binding capacity of specific IgE may range from 50–150%. Considering that total IgE binding of a birch pollen extract is mainly caused by Bet v 1, we cover at least the 50–100% range which is represented by accuracies of > 0.48 (MR) to < 1.21 (IB) at 40% rBet v 1a to 1.0 at 100% rBet v 1a (cf. Fig 7). Thus, deviations of the content of the actual active (i.e. IgE binding) component(s) in the allergen product of down to a factor of 0.5 are detected with reasonable accuracy of all assays used in this study.

Recombinant Bet v 1 has been generated as low IgE-binding (hypoallergenic) variants to test therapeutic potential (reviewed by Grönlund and Gafvelin, 2010). Reduced IgE binding capacity in *in vitro* assays is one parameter that qualifies a rBet v 1a variant as potential hypoallergenic candidate molecule for AIT. In this regard the immunoassays used in this study are well suited to screen for potential therapeutics as they easily detected IgE responses of ≤ 1% of total IgE binding.

Considering the potential use of recombinant Bet v 1a variants for AIT or as diagnostic reagents, the variants rBet v 1a and rBet v 1_S112P/R145P_ used in this study could be used as quality reference standards. However other attempts to assess Bet v 1-type protein conformation and thus potential allergenicity could also be considered to evaluate the quality of recombinant Bet v 1 preparations. The recently identified natural ligand of Bet v 1a and the determined structures of Bet v 1 isoforms with bound ligands could be employed for the development of quantitative ligand binding assays with clear-cut correlation to allergen conformation und thus IgE binding activity [5,55]. Such applications would clearly advance the evaluation of allergen folding-dependent IgE binding of rBet v 1 preparations.

The list of methods to analyze Bet v 1 conformation and correlated IgE interaction is not complete in this study. Surface plasmon resonance, infrared spectroscopy, isothermal titration calorimetry and other methods are also valuable additions to be included in a set of assays to assess the quality of recombinant Bet v 1-type allergens. However, lower IgE binding might not always be necessarily related to loss of Bet v 1-type conformation. Additional preparation/storage-dependent phenomena like modifications of single amino acid side chains (e.g. deamidation, oxidation and others) while maintaining native protein fold may also contribute to decreased IgE binding due to decreased antibody affinity on the epitope level.

Taken together the set of physico- and immunochemical assays used here to correlate rBet v 1a protein conformation and IgE binding revealed that ELISA and Circular Dichroism could accurately and precisely determine lower rBet v 1a contents (≤ 60%) in the context of absolute allergen concentration whereas immunoblot and cellular mediator release needed larger deviations of rBet v 1a ratios, ≥ 20% and ≥ 1%, respectively, to resolve the content of IgE-binding rBet v 1a in recombinant protein preparations. The principal findings in this study should be considered when employing methods for quality assurance of allergen preparations used in *in vivo* diagnostics and allergen-specific immunotherapies.

## Supporting Information

S1 File ^15^N spin relaxation of rBet v 1a. (PDF)

S1 Table Summary of circular dichroism of rBet v 1a protein combinations. (PDF)

S2 Table Experimental and theoretical values of quantitative IgE immunoassays. (PDF)
