# Supplementary material for: Folded or Not? Tracking Bet v 1 Conformation in Recombinant Allergen Preparations
Source: PLoS One. 2015 Jul 17;10(7):e0132956. doi: 10.1371/journal.pone.0132956 (PMC4506129; doi:10.1371/journal.pone.0132956)
Supplement: S1 File — (PDF) [file pone.0132956.s001.pdf]

Supplemental data-Figure S1

Folded or not? Tracking Bet v 1 conformation in recombinant allergen preparations

Felix Husslik[1*], Kay-Martin Hanschmann[2*], Ariane Krämer[1*], Christian Seutter von Loetzen[3], Kristian Schweimer[3], Iris Bellinghausen[4], Regina Treudler[5], Jan C. Simon[5], Lothar Vogel[1], Elke Völker[1], Stefanie Randow[1], Andreas Reuter[1], Paul Rösch[3], Stefan Vieths[1], Thomas Holzhauser[1], and Dirk Schiller[1].

*contributed equally

[1]Division of Allergology, Paul-Ehrlich-Institut, 63225 Langen, Germany.

[2]Section Biostatistics, Paul-Ehrlich-Institut, 63225 Langen, Germany.

[3]Department of Biopolymers, University of Bayreuth, 95440 Bayreuth, Germany

[4]Department of Dermatology, University Medical Center, 55131 Mainz, Germany

[5]Klinik für Dermatologie, Venerologie und Allergologie, Universität Leipzig, 04103 Leipzig, Germany

Results

To analyze the oligomerization state of Betv1a ^{15}N spin relaxation experiments were performed. The relaxation rates depend on the molecular tumbling in solution and are well suited parameters for investigation of hydrodynamic properties and therefore of the oligomerization state. At 14.1 T magnetic field strength and 298 K sample temperature the average transversal relaxation rate (R_2) of ^{15}N backbone amide nitrogens is 11.5 ± 1.1 s^{-1}, and the average longitudinal relaxation rate (R_1) is 1.29 ± 0.06 s^{-1}. The rotational correlation time of Betv1a for a simple isotropic model determined from the relaxation data is 9.04 ns, a typical value for a globular protein in the 15-20 kDa range protein [1]. This characterizes Betv1a as a monomeric protein under the high concentration conditions required for NMR spectroscopy.

Material and Methods

The preparation of ^{15}N labeled Bet v 1a [2] and the sequence-specific assignment of the amide resonances of Bet v 1a are reported elsewhere [3]. ^{15}N longitudinal (R_1) and transversal (R_2) relaxation rates were determined using standard methods at 14.1 T magnetic field strength using a sample with 0.5 mM ^{15}N labeled Betv1a in 20 mM sodium phosphate, pH 7.0 at 298 K. The rotational correlation time for an isotropic model was determined from the relaxation rates with the tensor2 program package [4].

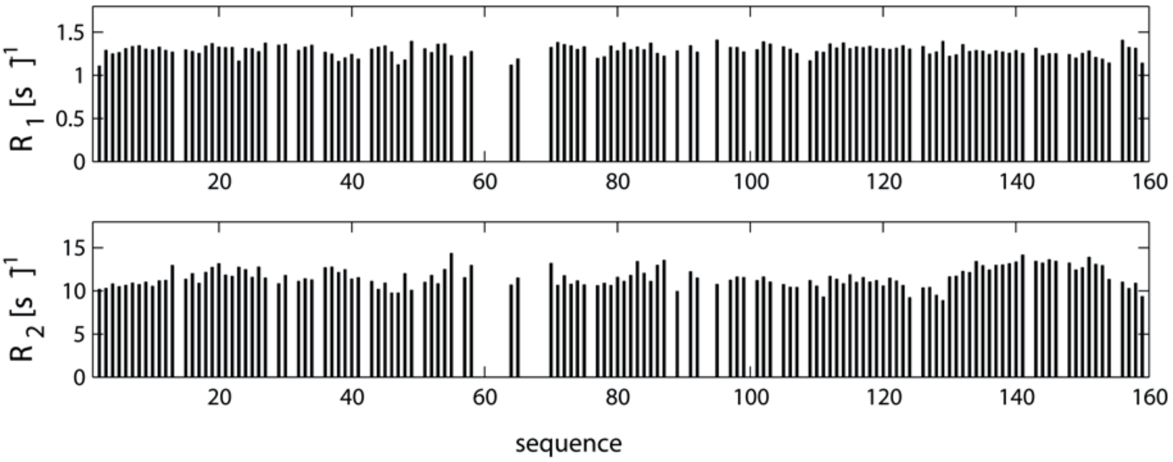

Figure_S1 ^{15}N spin relaxation data (Longitudinal (R_1, top) and transverse (R_2, bottom) relaxation rate) of Bet v 1a at 14.1 T magnetic field strength and 298 K.

References

1. Rossi P, Swapna GV, Huang YJ, Aramini JM, Anklin C, Conover K, et al. A microscale protein NMR sample screening pipeline. J Biomol NMR. 2010;46: 11-22.

2. Seutter von Loetzen C, Hoffmann T, Hartl MJ, Schweimer K, Schwab W, Rösch P, et al. Secret of the major birch pollen allergen Bet v 1: identification of the physiological ligand. Biochemical Journal. 2014;457: 379-390.

3. Schweimer K, Sticht H, Nerkamp J, Boehm M, Breitenbach M, Vieths S, et al. NMR Spectroscopy Reveals Common Structural Features of the Birch Pollen Allergen Bet v 1 and the Cherry Allergen Pru a 1. Appl Magn Reson. 1999;17: 449-456.

4. Dosset P, Hus JC, Blackledge M, Marion D. Efficient analysis of macromolecular rotational diffusion from heteronuclear relaxation data. J Biomol NMR. 2000;16: 23-28.
