# Supplementary material for: Folded or Not? Tracking Bet v 1 Conformation in Recombinant Allergen Preparations
Source: PLoS One. 2015 Jul 17;10(7):e0132956. doi: 10.1371/journal.pone.0132956 (PMC4506129; doi:10.1371/journal.pone.0132956)
Supplement: S1 Table — (PDF) [file pone.0132956.s002.pdf]

Supplemental Table 1: Summary of circular dichroism of rBet v 1a protein combinations

Bet v 1a ($_{S112P/R145P}$) [%]	λ [nm]	mean Θ	mean estimate (experimental)	95% CI	p-value	mean estimate (theoretical)	experimental /theoretical comparison
**0**	193	6292	10978	3035-9549	--	10978	1
**20**	193	3725	8411	468-6982	0.2587	8782	0.96
**40**	193	1398	6084	-1859-4655	0.0384	6587	0.92
**60**	193	-785	3901	-4041-2472	0.0044	4391	0.89
**80**	193	-2666	2020	-5923-591	0.0006	2196	0.92
**90**	193	-3689	997	-6946--432	0.0002	1098	0.91
**99**	193	-4821	-135	-8077--1564	<.0001	110	-1.23
**99.9**	193	-4966	-280	-8223--1709	<.0001	11	-25.5
**99.99**	193	-5316	-630	-8573--2059	<.0001	1.1	-574
**100**	193	-4686	--	-7943--1429	--	--	--
**0**	195	6423	13282	2297-10549	--	13282	1
**20**	195	3412	10271	-714-7538	0.2946	10625	0.97
**40**	195	475	7334	-3651-4601	0.0461	7969	0.92
**60**	195	-2074	4784	-6201-2052	0.0065	5313	0.90
**80**	195	-4733	2125	-8860--607	0.0007	2656	0.8
**90**	195	-6129	730	-10255--2003	0.0002	1328	0.55
**99**	195	-7195	-337	-11322---3069	<0.0001	133	-2.54
**99.9**	195	-7198	-340	-11325--3072	<0.0001	13.3	25.6
**99.99**	195	-7815	-956	-11941--3689	<0.0001	1.33	-720
**100**	195	-6859	--	-10985--2732	--	--	--
**0**	200	3921	14916	-285-8126	--	14916	1
**20**	200	582	11577	-3623-4787	0.2553	11933	0.97
**40**	200	-2590	8406	-6795-1615	0.0335	8950	0.94
**60**	200	-5630	5365	-9835--1425	0.0032	5966	0.9
**80**	200	-8563	2433	-12768--4358	0.0003	2983	0.82
**90**	200	-10049	946	-14255--5844	<0.0001	1492	0.63
**99**	200	-11354	-359	-15560--7149	<0.0001	149	-2.4
**99.9**	200	-11136	-140	-15341--6930	<0.0001	14.9	-9.41
**99.99**	200	-11709	-713	-15914--7504	<0.0001	1.49	-478
**100**	200	-10995	--	-15200--6790	--	--	--
**0**	218	-7521	3801	-8245--6797	--	3801	1
**20**	218	-6681	3961	-7405--5957	0.1027	3041	0.97
**40**	218	-6098	2378	-6822--5374	0.0089	2280	1.04

**60**	218	-5223	1503	-5947--4499	0.0001	1520	0.99
**80**	218	-4458	738	-5182--3734	<0.0001	760	0.97
**90**	218	-4048	328	-4772--3324	<0.0001	380	0.86
**99**	218	-3648	-72	-4372--2924	<0.0001	38	-1.89
**99.9**	218	-3696	-24	-4420--2973	<0.0001	3.8	-6.22
**99.99**	218	-3882	162	-4606--3158	<0.0001	0.38	426
**100**	218	-3720	--	-4444--2996	--	--	--
**0**	222	-6958	3838	-7619--6297	--	3838	1
**20**	222	-6090	2970	-6751--5429	0.067	3070	0.97
**40**	222	-5496	2375	-6157--4835	0.0039	2303	1.03
**60**	222	-4644	1524	-5305--3983	<0.0001	1535	0.99
**80**	222	-3901	781	-4562--3240	<0.0001	768	1.02
**90**	222	-3487	367	-4148--2826	<0.0001	384	0.96
**99**	222	-3197	77	-3858--2536	<0.0001	38	2
**99.9**	222	-3194	74	-3855--2533	<0.0001	3.8	19.3
**99.99**	222	-3318	198	-3979--2657	<0.0001	0.38	515
**100**	218	-3120	--	-3781--2459	--	--	--
