# Supplementary material for: Folded or Not? Tracking Bet v 1 Conformation in Recombinant Allergen Preparations
Source: PLoS One. 2015 Jul 17;10(7):e0132956. doi: 10.1371/journal.pone.0132956 (PMC4506129; doi:10.1371/journal.pone.0132956)
Supplement: S2 Table — (PDF) [file pone.0132956.s003.pdf]

Supplemental Table 2: Experimental and theoretical values of quantitative IgE immunoassays

Immunoblot

Bet v 1a ($_{S112P/R145P}$) [%]	mean estimate (experimental) in [au]	95% CI	p-value	mean estimate (theoretical) in [au]	experimental/theoretical comparison
**0**	$1.17*10^7$	$6.56*10^6$-$2.1*10^7$	--	$1.17*10^7$	1
**20**	$8.86*10^6$	$4.95*10^6$-$1.58*10^7$	0.0088	$9.39*10^6$	0.94
**40**	$6.88*10^6$	$3.85*10^6$-$1.23*10^7$	0.0001	$7.04*10^6$	0.98
**60**	$5.7*10^6$	$3.19*10^6$-$1.02*10^7$	<0.0001	$4.69*10^6$	1.21
**80**	$3.47*10^6$	$1.94*10^6$-$6.21*10^6$	<0.0001	$2.35*10^6$	1.48
**90**	$2.04*10^6$	$1.14*10^6$-$3.66*10^6$	<0.0001	$1.17*10^6$	1.74
**99***	$2.55*10^4$	$1.43*10^4$-$4.57*10^4$	<0.0001	$1.17*10^5$	0.22
**99.9**	$4.72*10^4$	$2.65*10^4$-$8.46*10^4$	<0.0001	$1.17*10^4$	4.03
**99.99**	$1.54*10^4$	$8.61*10^3$-$2.75*10^4$	<0.0001	$1.17*10^3$	13.16
**100**	$1.56*10^4$	$8.75*10^3$-$2.8*10^4$	<0.0001	--	--

Inhibition ELISA

Bet v 1a ($_{S112P/R145P}$) [%]	experimental EC_{50} [ng/well]	95% CI	p-value	theoretical EC_{50} [ng/well]	experimental/theoretical comparison
**0**	2.41	1.97-2.96	--	2.41	1
**20**	3.37	2.75-4.15	0.588	3.01	1.09
**40**	3.75	3.05-4.62	0.412	4.02	0.93
**60**	5.99	4.83-7.44	0.182	6.03	0.99
**80**	10.46	8.32-13.2	0.039	12.05	0.87
**90**	17.31	13.55-22.2	0.093	24.1	0.72
**99**	144.45	109.57-190.73	0.724	241	0.61
**99.9**	691.05	503.35-954.87	0.002	2410	0.29

**99.99**	no slope	--	--	24100	--
**100**	no slope	--	--	--	--

Mediator release (RBL)

**Bet v 1a ($_{S112P/R145P}$) [%]**	**experimental EC_{50} [ng/well]**	**95% CI**	**p-value**	**theoretical EC_{50} [ng/well]**	**experimental/theoretical comparison**
**0**	0.68	0.4-1.17	--	0.68	1
**20**	0.73	0.43-1.25	0.995	0.85	0.86
**40**	0.86	0.51-1.51	0.984	1.13	0.76
**60**	0.81	0.48-1.4	0.951	1.7	0.48
**80**	0.9	0.53-1.58	0.902	3.4	0.26
**90**	2.05	1.08-3.87	0.936	6.8	0.3
**99**	89.76	44.4-174.8	0.682	68	1.32
**99.9**	no slope	--	--	680	--
**99.99**	no slope	--	--	6800	--
**100**	no slope	--	--	--	--
